# Regular dental visits, periodontitis, tooth loss, and atherosclerosis: The Ohasama study

**DOI:** 10.1111/jre.12990

**Published:** 2022-04-06

**Authors:** Sho Yamada, Takamasa Komiyama, Takashi Ohi, Takahisa Murakami, Yoshitada Miyoshi, Kosei Endo, Takako Hiratsuka, Azusa Hara, Michihiro Satoh, Yukako Tatsumi, Ryusuke Inoue, Kei Asayama, Masahiro Kikuya, Atsushi Hozawa, Hirohito Metoki, Yutaka Imai, Takayoshi Ohkubo, Yoshinori Hattori

**Affiliations:** ^1^ Division of Aging and Geriatric Dentistry Department of Rehabilitation Dentistry Tohoku University Graduate School of Dentistry Sendai Japan; ^2^ Japanese Red Cross Ishinomaki Hospital Ishinomaki Japan; ^3^ Division of Public Health, Hygiene and Epidemiology Faculty of Medicine Tohoku Medical and Pharmaceutical University Sendai Japan; ^4^ Division of Drug Development and Regulatory Science Faculty of Pharmacy Keio University Tokyo Japan; ^5^ Department of Hygiene and Public Health Teikyo University School of Medicine Tokyo Japan; ^6^ Department of Medical Information Technology Center Tohoku University Hospital Sendai Japan; ^7^ Tohoku Institute for Management of Blood Pressure Sendai Japan; ^8^ Department of Preventive Medicine and Epidemiology Tohoku Medical Megabank Organization Tohoku University Sendai Japan

**Keywords:** alveolar bone loss, atherosclerosis, epidemiology, periodontitis, regular dental visit, tooth loss

## Abstract

**Objective:**

We aimed to explore the association between regular dental visits and atherosclerosis and between periodontitis, number of remaining teeth, and atherosclerosis among community dwellers in Japan.

**Background:**

Few studies have examined the association between regular dental visits, periodontitis, tooth loss, and atherosclerosis in community dwellers in Japan.

**Methods:**

The participants of this cross‐sectional study included community dwellers aged ≥55 years and residing in Ohasama. Exposure variables were regular dental visits; periodontitis, defined as radiographic alveolar bone loss (BL); the Centers for Disease Control/American Academy of Periodontology (CDC/AAP) classification; and number of remaining teeth. The primary outcome was atherosclerosis, defined as maximum carotid intima‐media thickness ≥1.1 mm or confirmation of atheromatous plaque.

**Results:**

Of 602 participants, 117 had atherosclerosis. In the multivariate model, compared to those with regular dental visits, the odds ratio (OR) (95% confidence intervals [CIs]) of atherosclerosis among those with the absence of regular dental visits was 2.16 (1.03–4.49). Regarding BL‐max, compared with those in the first quartile, ORs (95% CIs) of those in the second, third, and fourth quartiles were 1.15 (0.65–2.30), 0.65 (0.32–1.35), and 1.57 (0.81–3.01), respectively. Regarding CDC/AAP classification, compared to those with no or mild periodontitis, ORs (95% CIs) for those with moderate and severe periodontitis were 2.48 (0.61–10.1) and 4.26 (1.01–17.5), respectively. Regarding the number of remaining teeth, compared to those with ≥20 teeth, ORs (95%CIs) for those with 10–19 and 1–9 teeth were 1.77 (1.004–3.12) and 0.96 (0.52–1.80), respectively.

**Conclusion:**

The absence of regular dental visits and presence of periodontitis are associated with atherosclerosis among community dwellers in Japan.

## 1 INTRODUCTION

Cardiovascular disease (CVD), which is one of the major non‐communicable diseases (NCDs), and its clinical manifestations (e.g., atherosclerosis) remain a major public health issue, and efforts are ongoing to decrease their incidence rates and cause‐specific mortality worldwide.^1^ In addition, CVD and atherosclerosis are also related to dementia, which is increasing in incidence worldwide.^2^, ^3^, ^4^, ^5^ For healthy living in older stages of life, it is necessary to prevent the incidence and aggravation of CVD and the associated clinical disease, atherosclerosis.

Several epidemiological studies have demonstrated that periodontitis, which is also considered an NCD, is associated with atherosclerosis and CVDs such as myocardial infarction or stroke.^6^, ^7^, ^8^ Ahn et al.^9^ revealed an association between clinical attachment loss, which was measured by radiographic alveolar bone loss (BL) and peripheral arterial disease. Further, regarding subclinical cardiovascular disease, several studies have reported that carotid intima‐media thickness (IMT) is related to periodontal disease.^10^, ^11^, ^12^, ^13^

In addition to periodontitis, several evidence‐based studies have indicated that masticatory performance and the number of remaining teeth, which are associated with nutritional status and dietary intake, and oral health behavior, which can prevent periodontitis and tooth loss, are associated with atherosclerotic disease.^14^, ^15^, ^16^, ^17^, ^18^ In this context, Sen et al.^19^ elucidated the relationship between regular dental visits and the incidence of stroke. Regular dental visits are essential for the maintenance of oral health, such as periodontal status and the number of remaining teeth. Therefore, it is intuitive that regular dental visits are associated with atherosclerosis and CVD. However, few studies have examined the association between regular dental visits and atherosclerosis among community dwellers in Japan. Exploring the simple oral indicator associated with atherosclerosis leads to subsequent dental treatment and/or any health intervention, which may subsequently reduce the risk of CVD.

The primary aim of this cross‐sectional study was to explore the association between regular dental visits and atherosclerosis among community‐dwelling middle‐aged and older adults in Japan. The secondary aim of this study was to explore the association between periodontitis, the number of remaining teeth, and atherosclerosis in the same population.

## 2 MATERIALS AND METHODS

### 2.1 Design and study participants

The present study was conducted as a part of the Ohasama study: a self‐measurement of blood pressure at home and ambulatory blood pressure monitoring project since 1986. The project targeted community dwellers aged ≥55 years in Japan. The details of this project have been described elsewhere.^20^, ^21^, ^22^, ^23^ Oral examinations have been conducted since 2005. In 2005, the total population of Ohasama town was 6585. Of these, 3182 were aged ≥55 years. From 2005 to 2016, a cumulative total of 1312 people participated in the Ohasama study and provided written informed consent. Of these, participants for whom data of on second or subsequent examinations (*n* = 477), edentulous individuals (*n* = 102), and those with missing data for oral health indicators (*n* = 109) and atherosclerosis (*n* = 16) were excluded. Finally, the analyses were performed on 602 individuals. This study was approved by the Institutional Review Board of the Tohoku University Tohoku Medical Megabank Organization (approval number: 2019‐4‐67) and Teikyo University School of Medicine (approval number: 16‐075‐6) in compliance with the 1964 Helsinki Declaration and its later amendments or comparable ethical standards.

### 2.2 Oral examination

Four well‐trained dentists examined the periodontal status and the number of remaining teeth. In preparation for examining the oral cavity, all researchers were calibrated to target examinees using a periodontal probe for calibration, and the senior clinical researcher (T.O.) instructed the other researchers regarding oral cavity examination techniques. During the research period, constant meetings were held to ensure inter‐and intra‐examiner reproducibility. Conducted by a questionnaire, regular dental visits were divided into regular (regular dental visits without any symptoms) and episodic (in cases of concern about oral status, inquiring about an oral problem, never going to the dental office for any symptoms) visits.

In this study, radiographic alveolar BL and the Centers for Disease Control/American Academy of Periodontology (CDC/AAP) classification were used as indicators of periodontitis.^24^ BL was measured for all remaining teeth on the mesial and distal sides, except for the third molar, using fine panoramic dental X‐ray equipment (Veraviewepocs X550, J Morita Mfg Corp) and digital image measurement software (Quick Grain Standard; Inotech). BL was defined as the ratio of the vertical distance from the cement–enamel junction to the anatomical root apex to from the alveolar bone crest to the cement–enamel junction, based on the Schei ruler method.^25^ Measurement for BL was conducted by a trained dentist. BL‐max, which was the highest score for each participant, was applied. Participants were divided into quartiles based on the BL‐max score (e.g., the lowest BL‐max score group was the first quartile). Regarding the CDC/AAP classification, periodontitis was divided into three groups: normal (no or mild periodontitis), moderate periodontitis, and severe periodontitis.^9^

The number of remaining teeth was defined as the total number of remaining teeth excluding residual roots and teeth with grade three mobility.

### 2.3 Atherosclerosis

Atherosclerosis was evaluated by carotid ultrasound using a real‐time B‐mode ultrasound imaging unit (Toshiba Sonolayer SSA‐250A; Toshiba) with a 7.5 MHz annular array probe. The participants were evaluated for carotid IMT and atheromatous plaques in a sitting position. The observation area of IMT included the near and far walls on both sides of the common carotid arteries, bifurcations, internal carotid arteries, and external carotid arteries evaluated by a trained doctor from three different angles (anterior, lateral, and posterior) and 10 mm from the carotid bifurcation to the central side. The observation area of atheromatous plaque included the proximal and distal walls of the left and right common carotid arteries, bifurcations, internal carotid arteries, and external carotid arteries, respectively. An atheromatous plaque was defined as a discrete protruded lesion with an inflection point in the observation area.^26^ IMT was defined as the highest IMT score in each area, except in areas with thickening caused by atheromatous plaque. Atherosclerosis was defined when max‐IMT, which was the maximum value of IMT between each area, was ≥1.1 mm, or when an atheromatous plaque was confirmed.^27^

### 2.4 Covariates

The present study surveyed body mass index (BMI), current medical history (diabetes and dyslipidemia), antihypertensive medication use, systolic blood pressure (SBP), diastolic blood pressure (DBP), smoking, drinking alcohol, and educational attainment. These covariates were selected based on previous epidemiological research focusing on regular dental visits, periodontitis, tooth loss, and atherosclerosis.^19^, ^26^ BMI (kg/m^2^) was divided into underweight (<18.5), 18.5–<25.0 (normal), and ≥25.0 (overweight).^28^ The definitions of diabetes and dyslipidemia were based on the diagnosis of these diseases, including information regarding current treatment. Home blood pressure data were collected using a semiautomatic BP measuring device (HEM401C; Omron Healthcare Co, Ltd). The participants were required to measure their BP in the morning and the evening for four weeks. Detailed methods to collect home blood pressure data are reported elsewhere.^29^ SBP (mm Hg) was categorized into <115, 115–124, 125–134, and ≥135, respectively, and DBP (mm Hg) was categorized into <75, 75–84, and ≥85, respectively. These scores referred to the Japanese Society of Hypertension Guidelines for the Management of Hypertension.^30^ Smoking and alcohol drinking statuses were divided into current, past, or no smoking/drinking. Educational attainment was defined as the number of years of education after elementary school, and the participants were grouped into those who received <10 years and ≥10 years of education after elementary school.

### 2.5 Statistical analyses

The exposure variables were regular dental visits, BL‐max (quartile), CDC/AAP classification (no or mild periodontitis, moderate periodontitis, severe periodontitis), and the number of remaining teeth (≥20 teeth, 10–19 teeth, and 1–9 teeth). The primary outcome was atherosclerosis (i.e., max‐IMT ≥1.1 mm or confirmation of atheromatous plaque). In the analysis regarding the CDC/AAP classification, the participants with ≤3 teeth were excluded. The characteristics according to atherosclerosis status were examined using the Wilcoxon rank‐sum test for continuous variables and Fisher's exact test for categorical variables. A logistic regression model was applied for statistical examination of the association between oral health variables and atherosclerosis, and the odds ratios (ORs) and 95% confidence intervals (CIs) were calculated. Three models were created for the analyses: Model 1 was a null model; Model 2 was adjusted for age and sex; and Model 3 was adjusted for age, sex, BMI, current medical history, current medications, SBP, DBP, smoking, alcohol consumption, and educational attainment. Sensitivity analysis was conducted for the statistical examination of the association between oral health and other atherosclerosis criteria (i.e., mean‐IMT ≥0.9 mm or confirmation of atheromatous plaque).^31^ Statistical analyses were conducted using JMP Pro Ver.15 (SAS Inc.). All analyses were performed as two‐tailed tests, and *p*‐values < .05 were considered statistically significant.

## 3 RESULTS

Of the 602 participants, 100 partook in regular dental visits, 306 had ≥20 remaining teeth, 149 were in the first quartile of BL‐max, 43 had no or mild periodontitis according to the CDC/AAP classification, and 117 had atherosclerosis. The presence or absence of atherosclerosis varied significantly with age, sex, BMI, SBP, antihypertensive medication use, regular dental visits, the CDC/AAP classification, and the number of remaining teeth (Table 1). The regularity of dental visits varied significantly with drinking, educational attainment, the CDC/AAP classification, the number of remaining teeth, and the presence of atherosclerosis (Table S1).

**TABLE 1:** Participant characteristics according to atherosclerosis status

Variables	Overall (*N* = 602)	Atherosclerosis		*p*‐Value
		Presence (*n* = 117)	Absence (*n* = 485)	
Age, mean ± SD	66.0 ± 7.3	70.8 ± 7.0	64.8 ± 6.9	<.01
Male, %	37.7	53.0	34.0	<.01
BMI, %				
<18.5	3.2	3.4	3.1	<.01
18.5–24.9	58.1	70.9	55.0	
≥25.0	38.7	25.6	41.9	
SBP, %				
<115 mm Hg	15.0	7.7	16.7	<.01
115–124 mm Hg	23.4	21.4	23.9	
125–134 mm Hg	29.2	23.1	30.7	
≥135 mm Hg	32.1	47.9	28.3	
DBP, %				
<75 mm Hg	52.8	59.0	51.3	.25
75–84 mm Hg	36.4	29.1	38.1	
≥85 mm Hg	10.5	12.0	10.1	
Antihypertensive medication, %	46.5	61.5	38.5	<.01
Diabetes, %	8.3	9.4	8.0	.58
Dyslipidemia, %	32.1	25.6	33.6	.12
Smoking, %				
Never	73.8	66.7	75.5	.43
Former	13.5	18.8	12.2	
Current	11.6	13.7	11.1	
Drinking, %				
Never	49.2	42.7	50.7	.21
Former	5.0	6.8	4.5	
Current	44.2	49.6	42.9	
<10 years of education, %	43.0	41.9	43.3	.84
Regular dental visit, %	16.6	10.3	18.1	.04
BL‐max quartile, %				
First (≤48.0%)	24.8	22.2	25.4	.55
Second (48.1%–57.3%)	24.9	26.5	24.5	
Third (57.4%–72.3%)	25.4	17.1	27.4	
Fourth (≥72.4%)	24.9	34.2	22.7	
CDC/AAP classification, %				
No or mild	7.7	2.8	8.9	.04
Moderate	47.6	42.5	48.8	
Severe	44.7	54.7	42.4	
Number of remaining teeth, %				
≥20	50.8	39.3	53.6	.01
10–19	25.9	34.2	23.9	
1–9	23.3	26.5	22.5	

Abbreviations: BL, bone loss; BMI, body mass index; DBP, diastolic blood pressure; SBP, systolic blood pressure; SD, standard deviation.

Table 2 shows the results of tests for an association between regular dental visits, BL‐max, CDC/AAP classification, the number of remaining teeth, and atherosclerosis. Compared with participants who regularly visited the dentist, ORs (95% CIs) of atherosclerosis among those with episodic dental visits were significantly higher in Model 1 and Model 3. Regarding BL‐max, compared with those in the first quartile, the ORs (95% CIs) of those in the second, third, and fourth quartiles were not significantly higher in any models. Regarding CDC/AAP classification, compared to those with no or mild periodontitis, the ORs (95% CIs) of participants with atherosclerosis were significantly higher in those with severe periodontitis in Model 1 and Model 3. Regarding the number of remaining teeth, compared to those with ≥20 teeth, the OR (95% CIs) for atherosclerosis was significantly higher in those with 10–19 teeth in Model 1 and Model 3. The analysis of the relationship between each covariate and atherosclerosis using the multiple logistic regression model revealed that the OR increased significantly with age. The OR was significantly lower for females than males (Table S2).

**TABLE 2:** Association of oral health indictors and atherosclerosis

Oral health indicators	Patients, *n*	Model 1	Model 2	Model 3
		OR (95%CI)		
Regular dental visit				
With	100	1.00	1.00	1.00
Without	502	1.94 (1.02–3.68)*	2.00 (0.94–4.00)	2.16 (1.03–4.49)*
BL‐max quartile			*p* for trend^†^ = .37	
First	149	1.00	1.00	1.00
Second	150	1.23 (0.69–2.20)	1.21 (0.64–2.28)	1.15 (0.65–2.30)
Third	153	0.71 (0.38–1.34)	0.63 (0.32–1.25)	0.65 (0.32–1.35)
Fourth	150	1.72 (0.96–3.00)	1.58 (0.86–2.91)	1.57 (0.81–3.01)
CDC/AAP classification			*p* for trend = .01	
No periodontitis or mild	43	1.00	1.00	1.00
Moderate	265	2.73 (0.81–9.20)	1.76 (0.50–6.48)	2.48 (0.61–10.1)
Severe	249	4.05 (1.21–13.6)*	2.58 (0.71–9.39)	4.26 (1.01–17.5)*
Number of remaining teeth			*p* for trend = .87	
≥20	306	1.00	1.00	1.00
10–19	156	1.95 (1.21–3.14)*	1.63 (0.97–2.74)	1.77 (1.004–3.12)*
1–9	140	1.61 (0.97–2.67)	1.05 (0.60–1.89)	0.96 (0.52–1.80)

Note

Model 1: Crude model; Model 2: Adjust for age and sex; Model 3: Adjusted for age, sex, BMI, current medical history (diabetes and dyslipidemia), antihypertensive medication, SBP, DBP, smoking, drinking, education history.

Abbreviations: BL, bone loss; CI, confidence interval; OR, Odds ratio.

*p < .05, **p < .01, ^†^ *p* for trend was calculated in Model 3.

## 4 DISCUSSION

The present cross‐sectional study examined the association of oral health indicators with atherosclerosis in community‐dwelling people aged ≥55 years. After adjusting for age, sex, BMI, current medical history, antihypertensive medication use, SBP, DBP, smoking, alcohol consumption, and educational attainment, regular dental visits were associated with a reduced presence of atherosclerosis, although the association was slightly attenuated in the sensitivity analysis (Table S3). Further, an additional multivariate logistic regression model with inverse probability of treatment weighting that was based on propensity score indicated that regular dental visits were still associated with atherosclerosis (average treatment effect = 0.121; *p *< .01, data not shown). BL‐max was not associated with atherosclerosis. However, the CDC/AAP classification was associated with atherosclerosis in the primary analysis and showed a trend with atherosclerosis in the sensitivity analysis. The number of remaining teeth showed a significant association with atherosclerosis; however, this association disappeared in the sensitivity analysis.

To the best of our knowledge, this is the first study to demonstrate an association between regular dental visits and atherosclerosis. The results of the present study are consistent with those of previous studies reporting a relationship between regular dental visits and stroke and cardiovascular disease.^19^, ^32^ A biological mechanism for this association with atherosclerosis is that oral health awareness and improvements in oral health behaviors resulting from regular dental visits attenuate the risk of periodontitis and tooth loss, which can contribute to controlling the progress of atherosclerosis. Previous studies have reported that regular dental visits are associated with higher oral health literacy^33^ and with a lower risk of periodontitis and tooth loss.^34^, ^35^ From another perspective, a previous study revealed that emergency dental visits are associated with lower overall health literacy and that those with irregular dental visits had little health knowledge.^36^ If dentists can successfully persuade patients to change their visitation patterns from irregular to regular through approaches such as explaining the merits of maintaining oral health and the relationship between oral health and several health outcomes, the overall health literacy may improve, and the risk of atherosclerosis may be attenuated through improved oral health literacy. Regardless, future, longitudinal research is required to explore the relationship between regular dental visits and atherosclerosis, including information regarding oral health and overall health awareness.

Regarding the indicators of periodontitis, BL‐max was not associated with atherosclerosis. In the present study, evaluations of BL‐max focused on partial alveolar BL, whereas Ahn et al. allocated the alveolar BL as clinical attachment loss.^9^ Hayashida et al.^37^ reported that clinical attachment loss was less correlated with atherosclerosis than was periodontal pocket depth because a high score of clinical attachment loss was possibly attributable to non‐inflammatory indicators, such as heavy toothbrushing pressure. Considering that inflammatory indicators such as proinflammatory cytokines mediate the relationship between periodontitis and atherosclerosis,^38^, ^39^, ^40^ indicators that can evaluate the current inflammatory status of periodontal tissue may detect the association with atherosclerosis better than would alveolar BL. The CDC/AAP classification evaluated by both periodontal pocket depth and clinical attachment loss was associated with atherosclerosis; in the sensitivity analysis, a trend between the two was confirmed (*p* for trend < .01). These results were similar to those of previous studies that reported an association between periodontitis evaluated by the CDC/AAP classification and carotid IMT scores.^41^ In the present study, as there were few participants with no or mild periodontitis, these participants were grouped into the same category.^9^ Therefore, the association of periodontitis severity based on the CDC/AAP classification and atherosclerosis was possibly underestimated.

The number of remaining teeth was associated with atherosclerosis in only the 10–19 teeth group, which is not consistent with the findings of previous studies.^15^, ^16^ The number of teeth is considered an indicator of past periodontitis or a surrogate indicator of masticatory performance. Deterioration of masticatory performance is related to atherosclerosis mediated by changes in nutritional intake or malnutrition.^42^ In this study, the presence of atherosclerosis was similar between participants with ≥20 teeth and those with ≤9 teeth; however, retention of 10–19 teeth was associated with the presence of atherosclerosis. As for the context in which the number of remaining teeth is a surrogate indicator of masticatory performance, the association between the number of remaining teeth and atherosclerosis does not seem to be robust. Given that among individuals with 10–19 teeth, 92.3% displayed moderate or severe periodontitis, as evaluated by the CDC/AAP classification, it appears that the inflammatory status of periodontal tissue, which depends on the number of remaining teeth, may be associated with atherosclerosis.

In this study, smoking, which has been previously observed to negatively affect atherosclerosis, was not associated with atherosclerosis.^43^ The Ohasama study has been conducted since 1986 and has provided data for subsequent medical consultations and accompanying health interventions by physicians, public health nurses, and municipalities. Indeed, the current smoking rate of the participants in the present study (11.6%) was lower than the rate among those in their 60s from the national health and nutritional survey in Japan (19.4%).^44^ One potential explanation for this discrepancy is that continuous public health attempts may have contributed to attenuating the association of smoking with atherosclerosis.

There are some limitations to this study. First, due to the cross‐sectional design, it is difficult to clarify whether regular dental visits precede the onset of atherosclerosis. Future research is expected to focus on the duration of regular dental visits, variation in the IMT score, or causal inference using prospective cohorts. Second, this study did not consider information regarding health awareness or physical activity, which are possible confounders. Although educational attainment is considered a surrogate indicator of health awareness and physical activity,^45^ these factors need to be considered directly. Third, the sample size was relatively small, making conducting multifaceted analyses, such as stratified analysis with age, sex, and other variables challenging. Future research with larger samples will help to elucidate the relationship between regular dental visits, periodontitis, the number of remaining teeth, and atherosclerosis. Finally, the population of this study was community dwellers in a limited area of Japan. Further studies from various populations are needed to confirm the generalizability of the current findings.

In conclusion, this cross‐sectional study in community‐dwelling people aged ≥55 years indicates that regular dental visits and periodontitis are associated with a reduced presence of atherosclerosis. Delineating information regarding the benefit of regular dental visits could help identify interventions that could reduce the prevalence of atherosclerosis.

## CONFLICT OF INTEREST

The authors have no conflicts of interest to declare.

## AUTHOR CONTRIBUTIONS

Takashi Ohi, Yutaka Imai, Takayoshi Ohkubo, and Yoshinori Hattori contributed to the study concept and design. Sho Yamada, Takamasa Komiyama, Takashi Ohi, Takahisa Murakami, Yoshitada Miyoshi, Kosei Endo, Takako Hiratsuka, Azusa Hara, Michihiro Satoh, Yukako Tatsumi, Ryusuke Inoue, Kei Asayama, Masahiro Kikuya, Atsushi Hozawa, Hirohito Metoki, Yutaka Imai, and Takayoshi Ohkubo were involved in data acquisition. Sho Yamada, Takamasa Komiyama, Takashi Ohi, Takahisa Murakami, Yoshitada Miyoshi, Kosei Endo, Takako Hiratsuka, and Yoshinori Hattori contributed to data analysis and interpretation. Sho Yamada and Takamasa Komiyama drafted the manuscript. Sho Yamada, Takamasa Komiyama, Takashi Ohi, Takahisa Murakami, Yoshitada Miyoshi, Kosei Endo, Takako Hiratsuka, Azusa Hara, Michihiro Satoh, Yukako Tatsumi, Ryusuke Inoue, Kei Asayama, Masahiro Kikuya, Atsushi Hozawa, Hirohito Metoki, Yutaka Imai, Takayoshi Ohkubo, and Yoshinori Hattori contributed to the critical revisions of the manuscript. All authors have read and approved the final manuscript.

## Supporting information

Table S1‐S3

## Data Availability

The authors elect to not share data.
