# Supplementary material for: Regular dental visits, periodontitis, tooth loss, and atherosclerosis: The Ohasama study
Source: J Periodontal Res. 2022 Apr 6;57(3):615–22. doi: 10.1111/jre.12990 (PMC9321748; doi:10.1111/jre.12990)
Supplement: Supplementary file 1 — Table S1‐S3 [file JRE-57-615-s001.docx]

**Supplementary Material**

Supplemental Table 1: Participant characteristics according to regular dental visit status

| Variables |  | Regular dental visits | | P-value |
| --- | --- | --- | --- | --- |
|  | Overall | Presence | Absence |  |
|  | (N=602) | (n=100) | (n=502) |  |
| Age, mean ± SD | 66.0±7.3 | 65.1±6.7 | 66.8±7.4 | 0.05 |
| Male, % | 37.7 | 45.0 | 36.3 | 0.11 |
| BMI, % |  |  |  | 0.46 |
| <18.5 | 3.2 | 5.0 | 2.8 |  |
| 18.5–24.9 | 58.1 | 54.0 | 58.8 |  |
| ≥25.0 | 38.8 | 41.0 | 38.3 |  |
| SBP, % |  |  |  | 0.95 |
| <115 mmHg | 15.0 | 16.0 | 14.7 |  |
| 115–124 mmHg | 23.4 | 23.0 | 23.5 |  |
| 125–134 mmHg | 29.2 | 27.0 | 29.7 |  |
| ≥135 mmHg | 32.1 | 34.0 | 31.7 |  |
| DBP, % |  |  |  | 0.92 |
| <75 mmHg | 52.8 | 53.0 | 52.8 |  |
| 75–84 mmHg | 36.4 | 38.0 | 36.1 |  |
| ≥85 mmHg | 10.5 | 9.0 | 10.8 |  |
| Antihypertensive medication, % | 46.5 | 47.0 | 53.0 | 0.91 |
| Diabetes, % | 8.3 | 7.0 | 8.6 | 0.58 |
| Dyslipidemia, % | 32.1 | 39.0 | 30.7 | 0.19 |
| Smoking, % |  |  |  | 0.93 |
| Never | 73.8 | 72.0 | 74.1 |  |
| Former | 13.5 | 14.0 | 13.4 |  |
| Current | 11.6 | 13.0 | 11.4 |  |
| Drinking, % |  |  |  | <0.01 |
| Never | 49.2 | 33.0 | 52.4 |  |
| Former | 5.0 | 6.0 | 4.8 |  |
| Current | 44.2 | 58.0 | 41.4 |  |
| <10 years of education, % | 43.0 | 26.0 | 46.4 | <0.01 |
| BL-max quartile, % |  |  |  | 0.67 |
| First (≤48.0%) | 24.8 | 25.0 | 24.7 |  |
| Second (48.1–57.3%) | 24.9 | 23.0 | 25.3 |  |
| Third (57.4–72.3%) | 25.4 | 30.0 | 24.5 |  |
| Fourth (≥72.4%) | 24.9 | 22.0 | 25.5 |  |
| CDC/AAP classification, % |  |  |  | 0.04 |
| No or mild | 7.7 | 12.0 | 6.8 |  |
| Moderate | 47.6 | 53.0 | 46.4 |  |
| Severe | 44.7 | 35.0 | 46.8 |  |
| Number of remaining teeth, % |  |  |  | <0.01 |
| ≥20 | 50.8 | 75.0 | 46.0 |  |
| 10–19 | 25.9 | 17.0 | 27.7 |  |
| 1–9 | 23.3 | 8.0 | 26.3 |  |
| Atherosclerosis, % | 19.4 | 12.0 | 20.9 | 0.04 |

SD, standard deviation

BMI, body mass index; SBP, systolic blood pressure; DBP, diastolic blood pressure; BL, bone loss

Supplemental Table2. Association of covariates with atherosclerosis

|  | Model |
| --- | --- |
| Variables | OR (95% CI) |
| Age (with an increase in age of one year) | 1.13 (1.09–1.16) ^*^ |
| Sex |  |
| Male | Reference |
| Female | 0.41 (0.26–0.64) ^**^ |
| BMI |  |
| 18.5-24.9 | Reference |
| <18.5 | 1.29 (0.39–4.25) |
| ≥25 | 0.57 (0.35-0.92) |
| SBP |  |
| <115 | Reference |
| 115-124 | 1.35 (0.57–3.16) |
| 125-134 | 0.88 (0.37-2.06) |
| ≥135 | 1.91 (0.85-4.27) |
| DBP |  |
| <75 | Reference |
| 75-84 | 0.68 (0.42–1.12) |
| ≥85 | 1.01 (0.49-2.10) |
| Antihypertensive medication |  |
| Yes | Reference |
| No | 0.74 (0.47-1.16) |
| Diabetes |  |
| Yes | Reference |
| No | 0.74 (0.39–1.42) |
| Dyslipidemia |  |
| Yes | Reference |
| No | 1.37 (0.83-2.26) |
| Smoking |  |
| Never | Reference |
| Former | 1.04 (0.51–2.12) |
| Current | 1.42 (0.66–3.03) |
| Drinking |  |
| Never | Reference |
| Former | 1.37 (0.50–3.77) |
| Current | 1.13 (0.65–1.94) |
| Educational attainment |  |
| ≥ 10 years | Reference |
| < 10 years | 0.74 (0.44–1.26) |

BMI, body mass index; SBP, systolic blood pressure; DBP, diastolic blood pressure

*P < 0.05,　**P < 0.01.

Each variable was adjusted for age and sex. Age was adjusted for sex; sex was adjusted for age.

Supplemental Table 3: Association of oral health indictors and atherosclerosis (mean IMT is ≥0.9 mm, or confirmation of atheromatous plaque)

| Oral health indicators | Participants | Model 1 | Model 2 | Model 3 |
| --- | --- | --- | --- | --- |
|  |  | OR (95% CI) | | |
| Regular dental visit | | | | |
| With | 100 | 1.00 | 1.00 | 1.00 |
| Without | 502 | 1.68 (0.92–3.08) | 1.66 (0.86–3.22) | 1.80 (0.89–3.64) |
| BL-max quartile p for trend=0.20 | | | | |
| First | 149 | 1.00 | 1.00 | 1.00 |
| Second | 150 | 1.53 (0.85–2.73) | 1.55 (0.82–2.95) | 1.69 (0.84–3.39) |
| Third | 153 | 0.92 (0.50–1.72) | 0.85 (0.43–1.68) | 0.97 (0.47–2.00) |
| Fourth | 150 | 1.96 (1.11–3.45)* | 1.88 (1.01–3.51)* | 1.87 (0.95–3.65) |
| CDC/AAP classification　　　　　　　　　　　　　　　　　　　　　p for trend=0.007 | | | | |
| No periodontitis or mild | 43 | 1.00 | 1.00 | 1.00 |
| Moderate | 265 | 2.05 (0.70–6.01) | 1.27 (0.39–4.12) | 1.50 (0.42–5.33) |
| Severe | 249 | 3.23 (1.11–9.41)* | 2.01 (0.63–6.43) | 2.82 (0.80–10.0) |
| Number of remaining teeth p for trend=0.63 | | | | |
| ≥20 | 306 | 1.00 | 1.00 | 1.00 |
| 10–19 | 156 | 1.59 (0.99–2.57) | 1.24 (0.73–2.10) | 1.30 (0.73–2.29) |
| 1–9 | 140 | 1.71 (1.05–2.78)* | 0.94 (0.54–1.67) | 1.00 (0.54–1.82) |

OR, odds ratio; CI, confidence interval

*: p<0.05, **: p<0.01

BL, bone loss; IMT, intima-media thickness; CDC/AAP, the Centers for Disease Control/American Academy of Periodontology

Model 1: Crude model

Model 2: Adjusted for age and sex

Model 3: Adjusted for age, sex, body mass index, current medical history (diabetes, dyslipidemia), antihypertensive medication, systolic blood pressure, diastolic blood pressure, smoking, drinking, education history
